# The effect of burial in containers filled with naturally occurring soil and mine tailings on decomposition: a porcine pilot study

**DOI:** 10.1007/s00414-025-03715-8

**Published:** 2026-01-20

**Authors:** Artem Vitalievich Maikov, Jolandie Myburgh, Craig Adam Keyes

**Affiliations:** 1 Department of Forensic Medicine and Pathology, School of Clinical Medicine, Faculty of Health Sciences, University of the Witwatersrand, Johannesburg, South Africa; 2 Department of Anatomy, School of Medicine, Faculty of Health Sciences, University of Pretoria, Pretoria, South Africa

**Keywords:** Forensic, Taphonomy, Soil, Post-mortem interval, Quartzite, Dolomite

## Abstract

Due to the complexity of the decomposition process and all the variables affecting it, there are many factors that have not been thoroughly examined. The impact of different soil types on decomposition of buried remains has received relatively little attention. This study sought to investigate the impact of three soil types on decomposition. Fourteen piglet carcasses were used to assess and compare the decomposition patterns and rates that take place in dolomite and quartzite (two soil types common in the city of Johannesburg, South Africa), as well as in tailings from gold mines (another common feature in southern Johannesburg). Each piglet was buried in a container filled with one of these soil types (four for Dolomite, five for quartzite and mine tailings) and was periodically partially exhumed to record Total Body Score progression as thermal energy (measured in Accumulated Degree Days) accumulated. Soil samples were also taken to record the soil’s water content and pH level. The decomposition of the dolomite and mine tailing-interred piglets was found to differ significantly, with the latter progressing more rapidly. Dolomite was also consistently the soil type with the lowest water content and the least pH fluctuation, while quartzite had the highest water content and the most significant changes in pH over the course of the experiment. While this study has experimental limitations, it provides novel results that will help forensic practitioners understand subterranean decomposition in South Africa, and possibly other countries with similar climates and soil types.

## Introduction

The impacts of temperature and necrophagous insect activity on decomposition are well documented in forensic science. Burial, however, dampens temperature fluctuations and restricts insect access. For example, at a depth of 1 m, daily temperature fluctuation is reduced by about 3 °C [1], and even a shallow 60 cm burial can completely exclude Dipteran activity [2]. Despite this, the effects of burial on decomposition remain comparatively under-researched and are at least as complex as subaerial (surface) decomposition. Findings from subaerial studies cannot simply be transferred to burial environments, which introduce additional variables that materially change the condition of remains while altering the variables that primarily affect subaerial remains; differences that forensic investigators must consider.

A prime example of high-impact subaerial decomposition variables is insect activity. Although insects struggle to reach a body once it is buried, colonisation may occur pre-burial and the development of eggs and larvae may continue afterward [3]. Burial slows larval development [4], yet sufficiently large maggot masses can generate heat that locally elevates temperature and accelerates decomposition [2].

Depth and soil conditions shape the thermal environment of a burial and, in turn, the rate of decomposition. Extremely shallow burials (~ 10 cm) mirror ambient surface temperature curves, whereas burials near 1 m reduce daily maximal temperatures and raise minimal temperatures by about 3 °C [1]. Soil moisture also matters: because water conducts heat, damp soils transmit environmental temperatures more effectively than dry soils, which insulate more strongly [5].

Two influential standardisations - total body score (TBS) and accumulated degree days (ADD) - have improved consistency in reporting decomposition. Megyesi et al. [6] introduced TBS to score decomposition-driven morphological change across the head/neck, torso, and limbs, offering a common sequence from death to skeletonization. However, TBS accuracy depends on the reference sample and context; the US-derived descriptors in Megyesi et al. [6] are most reliable for comparable subaerial conditions. Megyesi et al. also examined total thermal energy exposure and found stronger correlations with TBS than with post-mortem interval (PMI). Thermal energy is represented as ADD - calculated as the sum of daily mean temperatures from deposition to discovery with a 0 °C base (temperatures below 0 °C treated as 0 °C) [2, 6] - correlates closely with TBS, enabling PMI estimates from local temperature records. A key advantage of ADD is cross-site comparability: it normalises for differing temperatures and exposure durations so that residual differences can be attributed to non-thermal environmental factors.

Many studies employ pigs as human analogues - not to produce directly interchangeable PMI estimates, but to ethically test decomposition variables on larger samples. Animal models also allow for a proof-of-concept study design to isolate important variables that can later be ethically validated on studies with small sample sizes of human remains [7]. Addressing this, Keough et al. [8] developed pig-based TBS tables for a research farm in Pretoria, South Africa. Adaptations exist for other contexts, such as total aquatic decomposition scores [9], yet no equivalent system has been established for buried remains.

In buried contexts, soil properties (particularly water content and pH) strongly influence rates and patterns of decomposition [10–13]. Too little water reduces microorganism motility; too much limits oxygen for aerobic organisms [10]. The effects of pH are nuanced: more acidic soils can accelerate decomposition compared with neutral or alkaline soils, provided water content and organic composition are similar [11]. Decomposition fluids further modify the local environment: while effects on soil moisture appear limited [12], pH typically rises initially [11] and then can drop substantially, becoming more acidic if buffering capacity is low [13].

Local geology underscores these dynamics. In Johannesburg, South Africa (the present study site), soils are predominantly dolomite in the northwest [14] and quartzite in the southeast [15]. Dolomite clays are alkaline, have low water retention, and high buffering and micronutrient capacity [16, 17]; quartzite-derived sandy loams are more acidic, retain more water, and have fewer micronutrients [16, 17]. The region’s historic gold mining has also created extensive mine tailings throughout the region – artificial sandy hills of fine, chemically treated waste material - often with extreme (typically acidic) pH and poor micronutrient content [18]. Pyrite in these tailings can oxidise to sulphuric acid during rainfall, mobilising heavy metals into groundwater [19]. Although revegetation is the usual remediation strategy, toxicity and nutrient loss hinder success [18]. The implications of such altered soils for decomposition of buried remains in South Africa have not been explored.

Given South Africa’s high homicide rate - about six times the global average [20, 21] - and the relative ease of concealing a body via shallow burial [22], a nuanced understanding of subterranean decomposition is essential for accurate PMI estimation and effective forensic investigation. Additionally, there is a significant dearth of information in the published literature on the effects of different soil types and their properties on the rate and patterns of decomposition and how it will influence forensic investigations.

To address this gap, this study examined decomposition rates and patterns using porcine analogues (*Sus scrofa domesticus*) buried in containers filled with three distinct soil types. Pigs were selected due to their anatomical and microbiome similarity to humans [23] and their accepted use as models in forensic taphonomy where human remains are unavailable or prohibited [7].

## Methodology

The sample included the carcasses of 14 piglets (*Sus scrofa domesticus*) donated by a commercial pig farm. The piglets were reported to have died of “red gut”, which is a generic term for some form of intestinal knotting, which is unlikely to impact the decomposition. No pigs were slaughtered for the purposes of this study. Since all piglets had the same recorded cause of death, this factor is not expected to skew the results. The piglets were frozen at the farm prior to collection and subsequently transferred to freezers at the Johannesburg Forensic Pathology Services Medico-Legal Mortuary (FPS MLL) for storage prior to the commencement of the experiment. The mortuary freezers are set to target − 5 °C; the temperatures used in the farm freezer were not noted. Stokes et al. [24] have showed that the freezing process does not lead to any significant changes in the patterns of decomposition for muscular tissue of *Sus scrofa domesticus* specimens, and this (along with the lack of other preservation methods) justified the use of freezers as storage prior to the commencement of the study. Due to the piglets becoming available earlier than anticipated, and before other logistics were established, the piglets were stored for four weeks prior to the commencement of the experiment. Only 14 were available at the time of collection, and no more were available from the farm thereafter, so it was decided that the dolomite sample would be made up of four piglets, while the other soils would have five.

This study used 14 65 L plastic containers with several small holes (diameter of 4 mm) drilled into the bottom of each (with a double layer of gauze to prevent loss of soil) to allow accumulated fluids and water to drain out of them. Each container was filled with 60 L of one of the soil types; four containers with dolomite (Soil D), five containers for quartzite (Soil Q), and five containers for mine tailings (Soil M). The soils were obtained from areas that were reported to have pure soil content [14, 15]. Dolomite was obtained from the Bolts Farm Quarry in the Sterkfontein region. Quartzite was obtained from West Park Cemetery in Albertville, Johannesburg. The mine tailing specimens were obtained from tailings owned by a private company wishing to remain anonymous, who donated samples from their tailings located at Westonaria, Gauteng. A single perinatal piglet was buried in each of these containers and partially exhumed at regular intervals for data gathering.

A secluded subaerial site at the FPS MLL was chosen as the site where the containers would be placed to prevent access by uninvolved parties. Previous research determined that there is no animal scavenging in this area [25]. This site was termed the primary site of research. Due to legislature regarding the transport and storage of potentially radioactive material (i.e. Mine tailings), research with this material was limited to the grounds belonging to the donor mining company, who facilitated the collection and disposal of the tailings used. The donor company provided the required health and safety training for work in their environment, as well as the co-signing of a Non-Disclosure Agreement governing the amount of information to be made publicly available. The facilities used were the workshops and surrounding areas located in Westonaria, Gauteng. This will hereafter be referred to as the secondary site of research. Both sites were uncovered and open to the elements and the containers were left without lids to simulate a shallow clandestine burial.

Each piglet was interred with one iButton Thermochron data logger (Cold Chain Thermodynamics) to record temperatures at two-hour intervals to calculate thermal energy. The accompanying software and hardware provided by Cold Chain Thermodynamics was used to retrieve the data. These data loggers were placed inside sealable plastic bags to prevent water damage. Another data logger (also within a sealable plastic bag) was placed above ground near the containers to record ambient temperatures. Four data loggers failed during the experiment; one buried in dolomite and one in mine tailings, as well as two buried in quartzite; however, this did not impede the study. Meteorological data was sourced from weather stations that make their data publicly available: station 0475879 0 (JHB BOT TUINE) for the mortuary temperatures and station 0475528B7 (ZUURBEKOM AWS) for the mining company workshop temperatures.

This study took place during the warm season of South Africa, from November 2021 to January 2022. Gauteng is a province in South Africa which experiences rainfall in summer with average temperatures exceeding 20 °C. Winters are dry and cold, with average temperatures near 10 °C and rare drops below 0 °C at night. The spring and autumn seasons were not deemed sufficiently distinct to warrant investigation.

Prior to interment, the TBS of each piglet was recorded according to the tables developed by Keough et al. [8]. A control sample of 100 g of soil from each container was gathered to compare against future specimens. A shovel was used to dig a hole 15 cm deep into each of the soil samples within the container, after which a piglet was placed into each one. Since depth of burial was not found to significantly affect decomposition [26], the depth was chosen to entirely cover the piglets, while not making regular exhumation too difficult. Each piglet was positioned on its side, with the data logger near its sternum. Thereafter, the piglets were buried in their corresponding soil types.

The average daily temperatures at both sites were recorded via publicly accessible meteorological data gathered at the aforementioned weather stations. When the sum of these average temperatures at either site reached a multiple of 50 ADD, the piglets at the corresponding site were partially exhumed so that one side of their body and two legs were visible to be scored using the TBS tables developed by Keough et al. [8]. Thereafter, the piglets were re-interred (i.e., the removed soil was replaced on top of the carcass). At every 100 ADD, a soil sample was taken from every specimen − 100 g of soil was collected ventral to the piglet, as close to its abdomen as possible. This method of soil collection is similar to that of DeBruyn et al. [27] and was chosen over collecting from beneath the body to minimise the disturbance to the carcass. This soil was stored in a Ziploc bag and labelled with the container it came from and the season, as well as the date and atmospheric ADD (e.g. D1 Warm; 06/11/2021; ADD 100). Photographs of the piglets were captured every 200 ADD for future examination and demonstration purposes.

Upon reaching 400 ADD, the frequency of exhumation was reduced from multiples of 50 ADD to multiples of 100 ADD. This was done to reduce the disturbance to the decomposition microbiome, and because this was noted by Myburgh et al. [22]. to be the beginning of a significant decline in the rate of decomposition. This study concluded when the cumulative thermal energy (as reported by the meteorological data) at the site reached 1200ADD. At this point, the piglets were flipped to observe their undersides before being placed in body bags to await incineration. Qualitative descriptions were also recorded for each piglet at every exhumation, and photographs were taken every 200ADD for future reference and comparison.

Soil samples which were gathered for subsequent testing were evaluated at the Humphrey Raikes Chemical Laboratory at the University of the Witwatersrand. At this laboratory, an oven, a volumetric flask, and a digital scale (OHAUS Digital scale, C.C Imelmann Laboratory Supplies) were used to determine the water content of the soil by gravimetric assessment. This process involved weighing out 50 g of the soil sample into a volumetric flask before placing it into a dry heat oven at 110 °C for 24 h and re-weighing it to determine the percentage mass that was water [28]. Deionised water, a pH meter (Eutech instruments, pH510), a magnetic stirrer/heater (FMH Instruments, C.C Imelmann Laboratory Supplies) with an accompanying stirring rod and volumetric flasks were used for the pH testing of the soil samples taken from the containers. This process used a 2:1 suspension-ratio of the soil (10 g of soil suspended in 20 ml of deionised water), which was then stirred for 30 min with a magnetic stirrer, with the mixture subsequently being tested with a digital pH meter to record the pH of the water [16]. Because this is a novel, proof-of-concept study, the soil properties that were investigated were limited to water content and pH. Additional soil samples have been retained by the authors for future studies to explore the potential of other soil properties not explored in this publication.

All data was analysed using Python and associated statistics libraries. Multiple Shapiro-Wilk tests were performed on the temperature and TBS data, and it was proven that all data was not normally distributed (α = 0.05 for this and all the subsequent tests). For this reason, non-parametric statistical tests were used throughout the analysis stage. Correlation between the reported meteorological data and data logger recordings was tested using Spearman correlation, and Mann-Whitney U (MWU) tests were used to test for significant differences. Two-sample Kolmogorov-Smirnov (KS) tests were used for the bulk of the comparisons. These tests were used to assess differences in temperature between the two research sites. KS tests were also used to compare the rates of decomposition between the soils (Dolomite – Quartzite, Dolomite – Mine tailing, and Quartzite – Mine tailing). Additionally, KS tests were used to determine if TBS-ADD progression of piglets within the same soil type was significantly different from each other.

## Results

### Effects of different research sites

**Fig. 1:**
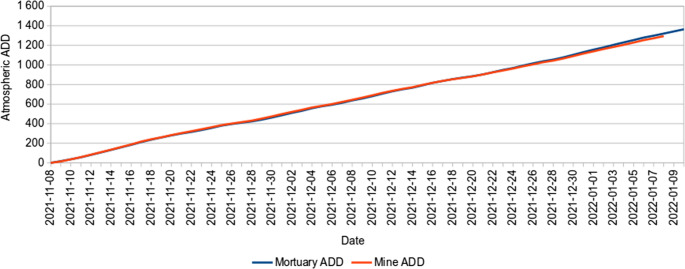
The rate of meteorological accumulated thermal energy (ADD) over the course of time at the mortuary and mine tailing workshop

Despite being situated approximately 70 km from each other, the temperatures reported by the weather stations showed that the sites had no significant differences, and an almost exact correlation (Fig. 1).

In contrast, the temperatures gathered by the data loggers differed significantly from the weather station data at both sites (MWU: *p* < 0.001 for both), being 2,5 °C higher than the weather station data, indicating that the effects of local microclimates, as well as the protective material covering the data loggers, played some role in altering the local temperatures. Despite this, the primary and secondary research sites’ data logger temperatures showed a high degree of correlation to the meteorological data (Spearman ρ = 0.83 and ρ = 0.85, respectively). This suggests that while the meteorologically reported and locally recorded data are significantly different, the general trends are similar enough that using meteorological data to schedule exhumation and data gathering is appropriate. The ADD recorded by the data loggers buried with the piglets was significantly greater than the ADD from the meteorological data (MWU: *p* < 0.001 in all cases). However, when comparing to the ADD recorded by the nearby data logger that was not buried, only one piglet recorded a significant difference.

### Intra-soil differences

Multiple KS tests were used to determine if the rate of decomposition of the specimens within the same soil type were significantly different from each other or not. The quartzite specimens were homogenous, with no significant differences between the rates of decomposition of the piglets (Table 1B). In contrast, all but one of the dolomite specimens had significantly different rates of decomposition from each other (Table 1A), with the same being true for the mine tailing specimens (Table 1C).

**Table 1:**
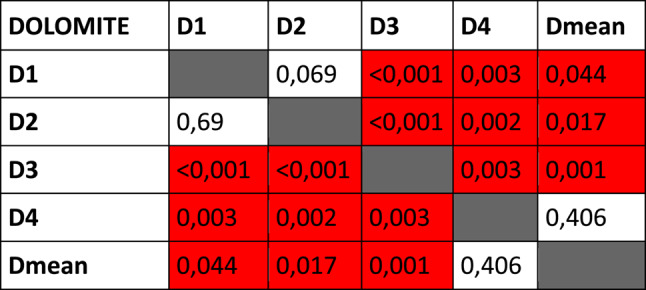

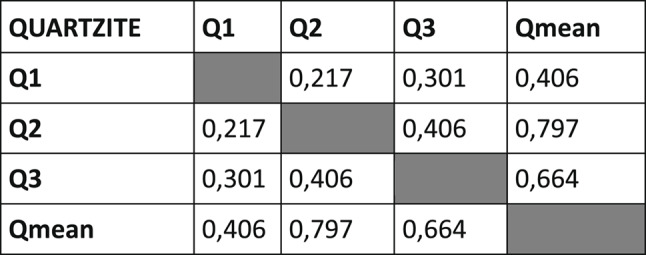

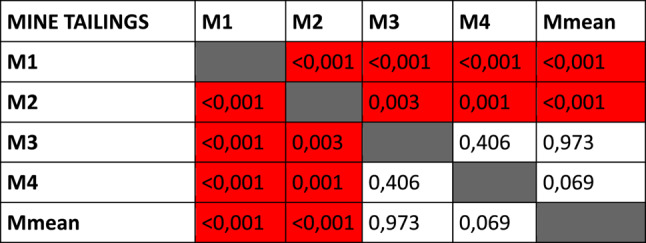
*P*-values of KS tests comparing changes in TBS of specimens within each soil type to each other. Significant differences highlighted in red

### TBS-ADD charts and equations

**Fig. 2:**
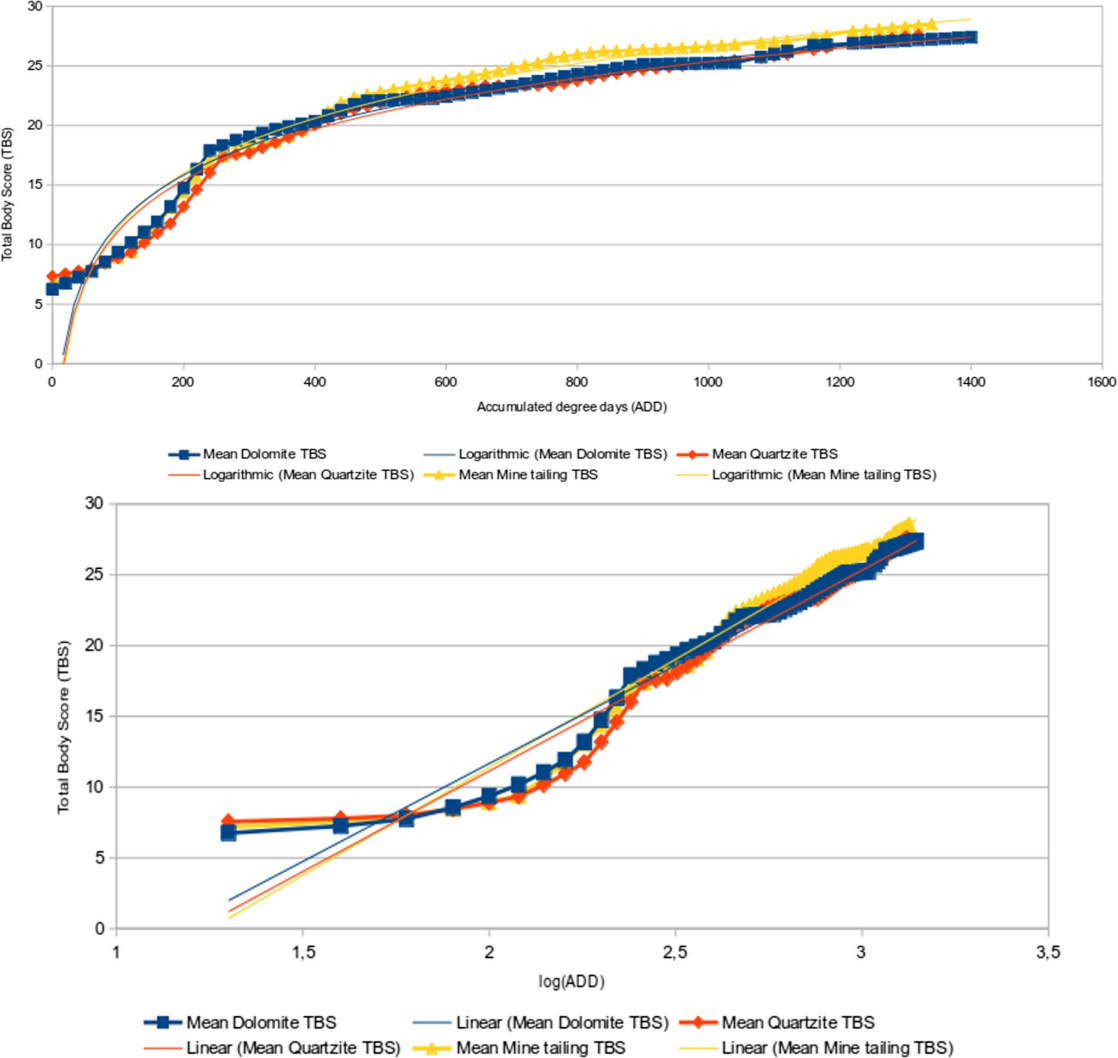
Mean TBS-ADD (top) and TBS(log10)-ADD (bottom) plots with equation trend lines

The mean TBS in each soil type was determined via linear interpolation, and these mean values were plotted on a single graph to visualise how different soil types affected the decomposition of the specimens (Fig. 2). The 10th log of the ADD was used to linearise the graph to simplify the equations for these graphs. Only the dolomite and mine tailing plots differed significantly from each other (KS: *p* = 0.046), indicating that in the other cases (dolomite – quartzite, quartzite – mine tailing), the rates of decomposition were too similar to be statistically distinguishable. The TBS-ADD graphs all had the general shape of a sigmoid curve, with an initial lagging phase, a subsequent sharp incline, and a final plateauing (Fig. 2).

The equations and correlation coefficients of each linearised plot are shown in Table 2. These equations show that the mine tailing specimens advanced to higher TBS the fastest, while the dolomite specimens advanced the slowest, suggesting that dolomite inhibits decomposition more than the mine tailing soil. The plots all have a high degree of correlation (R² ≥ 0.95 in all cases), which would suggest that the generated equations have very few outliers in establishing links between TBS and ADD.

**Table 2:** Linear equations and correlation coefficients of mean TBS-Soil log10(ADD) plots, where log10(ADD) is the independent variable

Soil type	Equation and correlation coefficient
Dolomite	$$\begin{array}{c}\mathrm f(\mathrm d)=13,8544510268923\;\mathrm d-16,039493661086\\R^2=0,967288893372574\end{array}$$
Quartzite	$$\begin{array}{c}\mathrm f(\mathrm q)=14,2112717417099\;\mathrm q-17,2893090670406\\R^2=0,948792372820444\end{array}$$
Mine tailing	$$\begin{array}{c}\mathrm f(\mathrm m)=15,2773894662853\;\mathrm m-19,156950485949\\R^2=0,957266621588847\end{array}$$

**Fig. 3:**
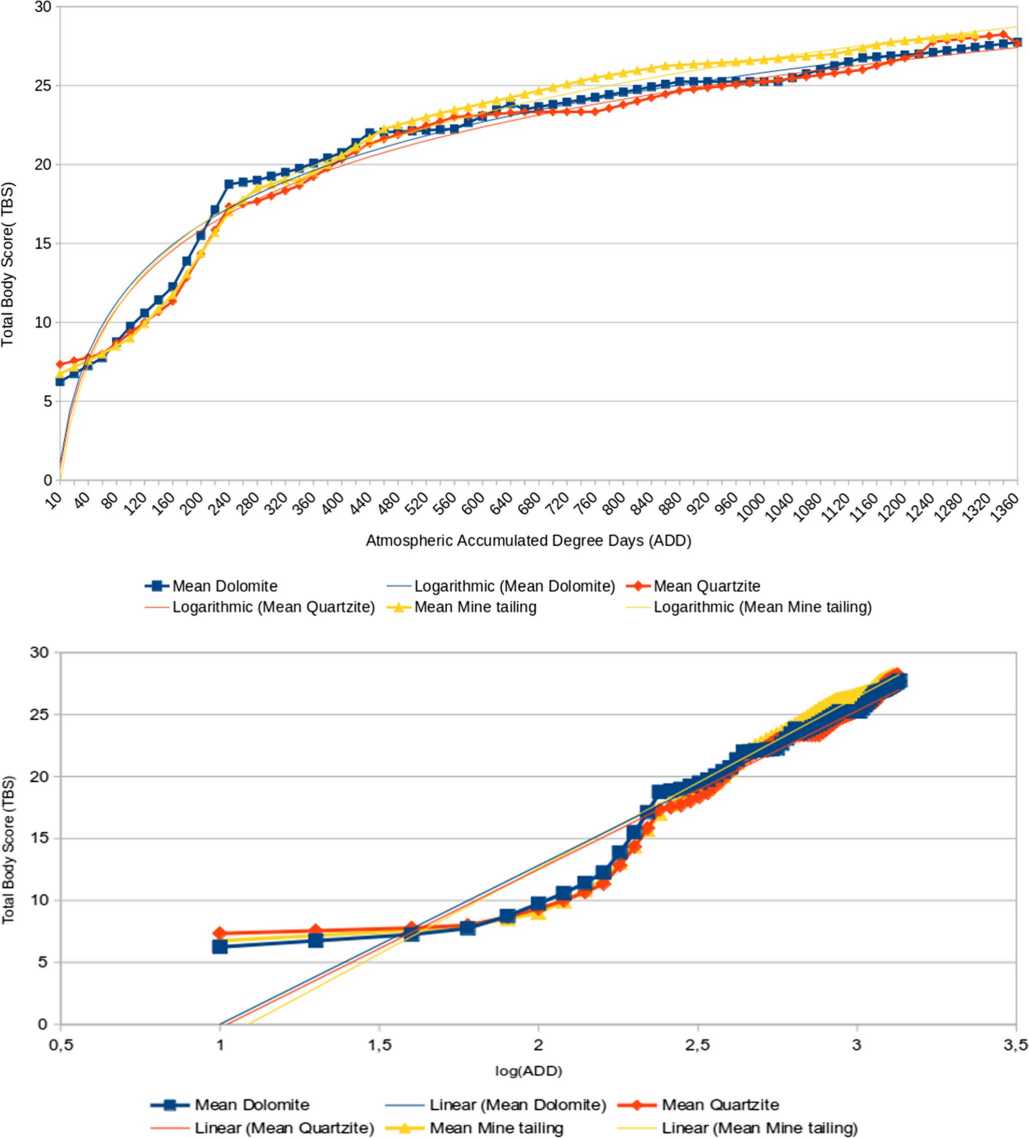
Mean TBS-atmospheric ADD (top) and TBS-log10(atmospheric ADD) (bottom) plots with equation trend lines

Since records of the ambient temperature of the air is more likely to be available than records of subterranean temperature during practical application of this data in an actual forensic case, trend lines for correlation between TBS and atmospheric ADD were also generated (Fig. 3), as well as the corresponding linear equations and correlation coefficients (Table 3). Mine tailing specimens are shown to have the fastest progression to higher TBS values, while Quartzite specimens had the slowest (although the difference between Quartzite and Dolomite specimens are marginal). This shows that decomposition progresses fastest in mine tailings and slowest in quartzite when tracking changes in TBS based on atmospheric temperature. The correlation coefficients are still high, but lower than what was observed with the soil ADD (R² ≥ 0.90 in all cases), suggesting a slightly higher deviation from the generated equations.

**Table 3:** Linear equations and correlation coefficients of mean TBS-Atmospheric log10(ADD) plots, where log10(ADD) is the independent variable

**Soil type**	**Equation and correlation coefficient**
Dolomite	f(d) = 12,8128483609638 d - 12,8000742545604 R^2^ = 0,949928200736925
Quartzite	f(q) = 12,789835286543 q - 3,0653571504684 R^2^ = 0,928796316266213
Mine tailing	f(m) = 13,8066677302769 m - 15,01356790189 R^2^ = 0,931816845413428

### Patterns of decomposition

Despite being buried, flies were observed near the containers at both sites throughout the early decomposition timeframe. Maggots were first observed in the mine tailing specimens (five days after burial), then in the quartzite specimens (10 days after burial), and lastly in the dolomite specimens (12 days after burial). Maggot activity also peaked much earlier in the mine tailing specimens than in either of the other soil types.

**Fig. 4:**
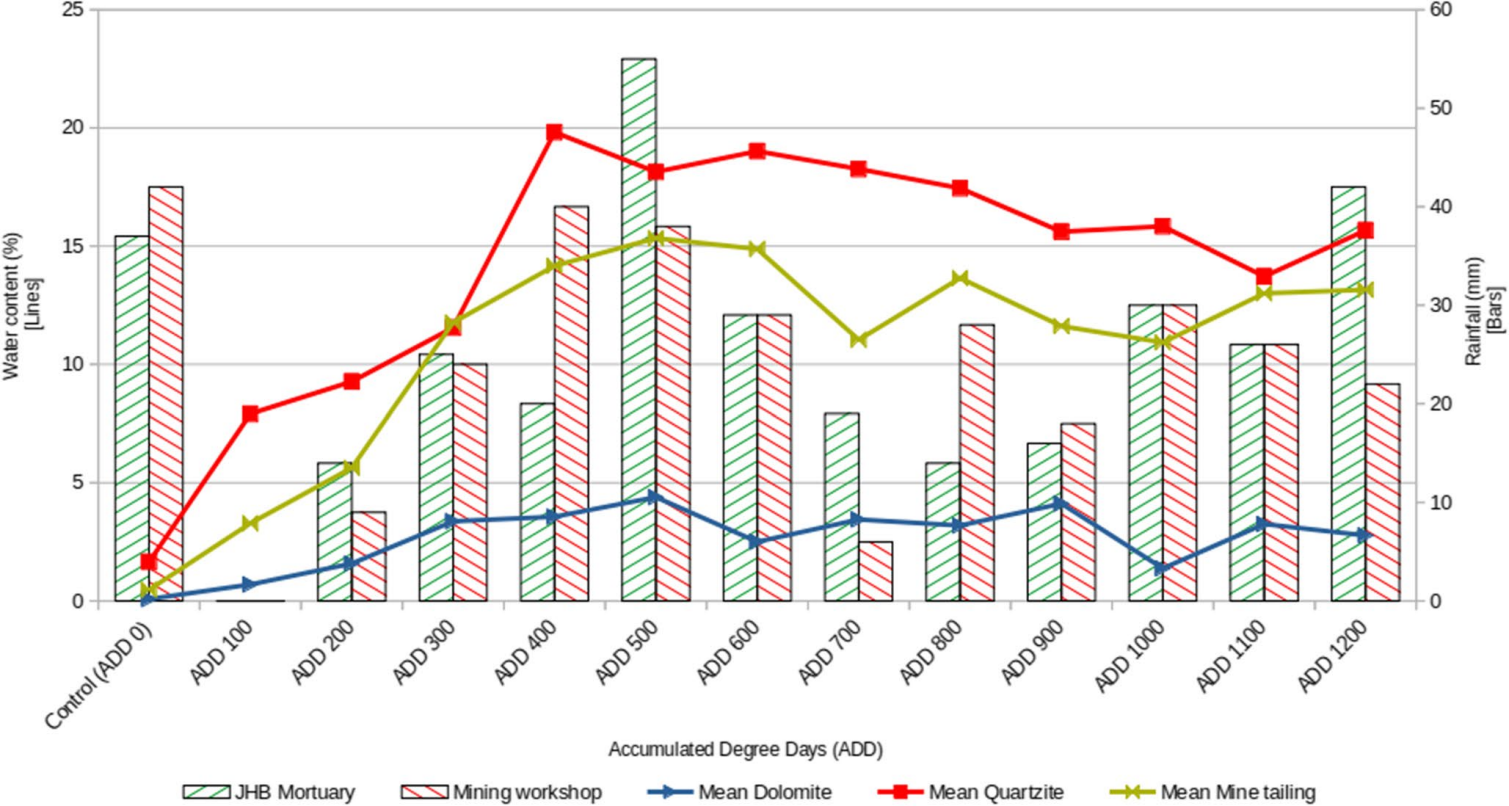
Soil water content (as a percentage of soil mass) shown as a line graph (left axis) and local rainfall in millimetres at both research sites shown as a bar graph (right axis)

Due to the frequent rains and high soil water content (Fig. 4), no mummification was observed as the piglets’ soft tissues were prevented from desiccating. Adipocere formation was noted on the mine tailing piglets 30 days after burial, with all piglets in each soil type eventually showing signs of saponification. The quartzite specimens were not observed to develop adipocere on their superior lateral sides, but upon being flipped at the conclusion of the experiment, most of them showed adipocere formation on the inferior lateral sides of their bodies. None of the dolomite-interred piglets formed any adipocere on either side of their bodies.

**Fig. 5:**
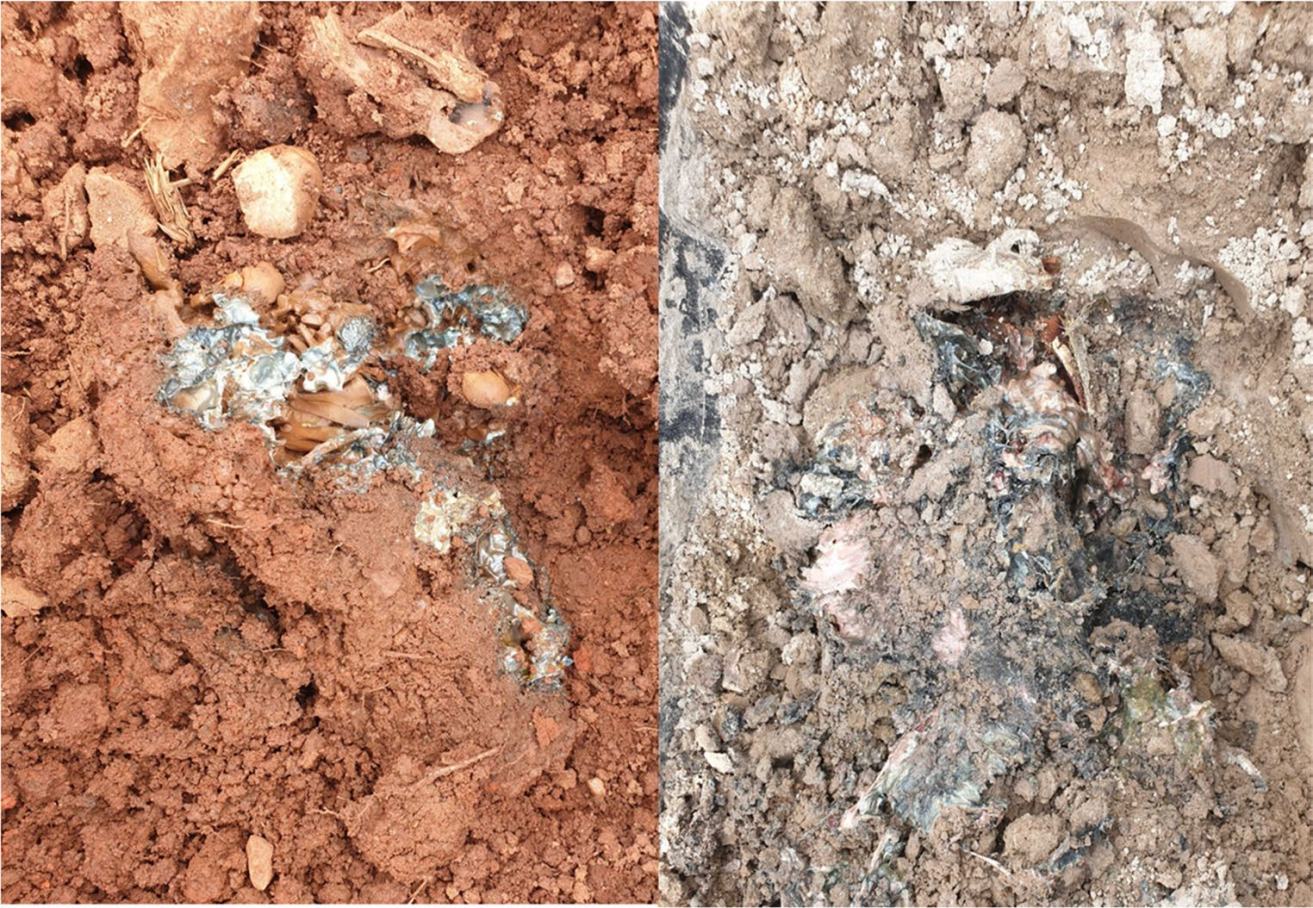
Adipocere formation visible on remains of piglet interred in quartzite (left) and mine tailings (right) after being flipped at conclusion of study

Due to the lack of preservation, the piglets’ remains were almost entirely consumed by maggots by the end of the data collection period, leaving only a small thoraco-abdominal tissue mass even in instances where saponification did occur (Fig. 5).

### Soil water content

Since the experiment was carried out outdoors, it was sometimes necessary to gather soil samples and make observations while it was raining. Because of this, some soil water content readings were much higher than would be suggested by the recorded rainfall.

Quartzite was found to retain water exceptionally well. The water content of quartzite noticeably exceeded that of the mine tailings and vastly exceeded that of the dolomite, despite being subjected to similar rainfall to the former, and identical rainfall to the latter (Fig. 4). Since high water retention has strong implications regarding the possibility of saponification and mummification, this is an important observation.

### Soil pH

**Fig. 6:**
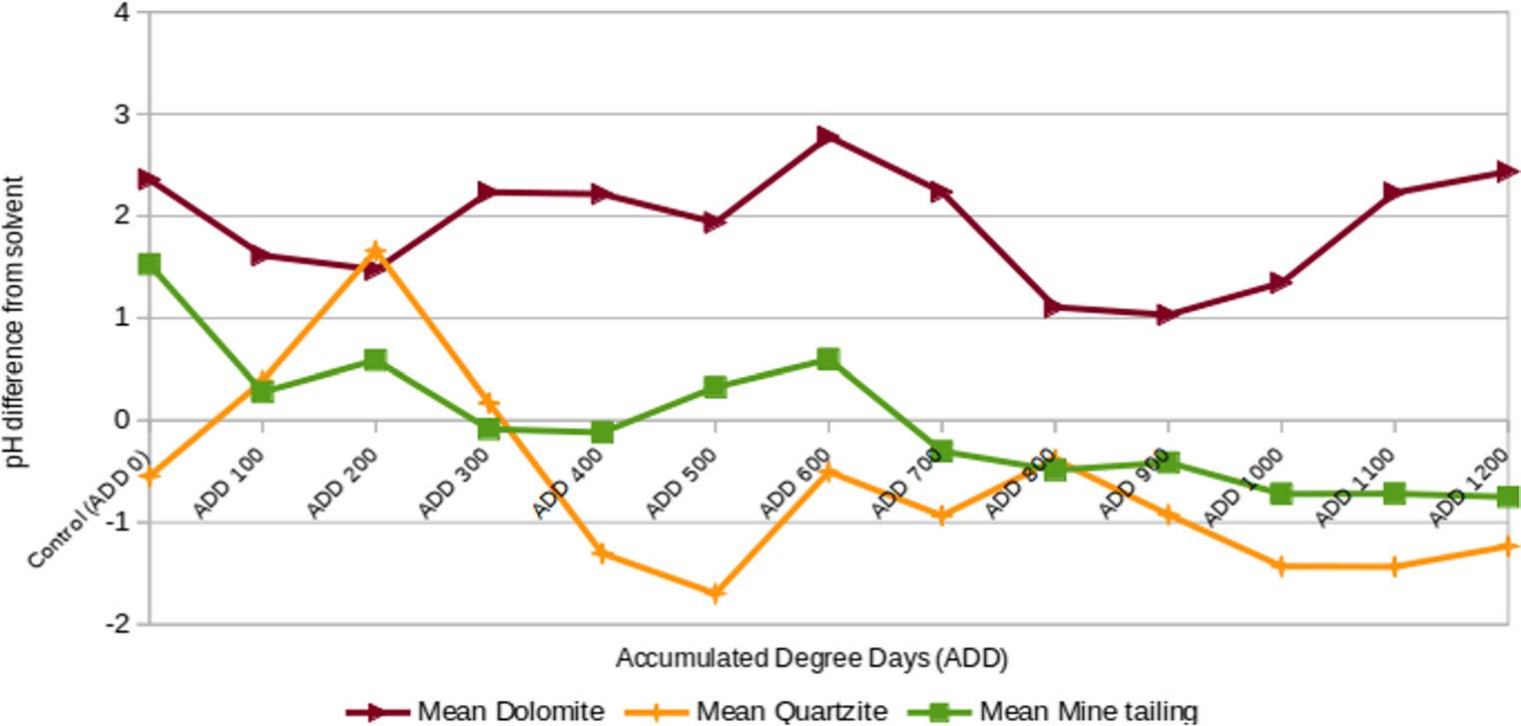
Mean soil pH, shown as difference from the pH of the solvent (deionised water)

The dolomite and mine tailing samples showed a slight amount of fluctuation until ADD600, at which point the pH of both decreased by one unit (mine tailing) or two (dolomite). The dolomite specimen then gradually returned to its initial pH, while the mine tailing remained more acidic (Fig. 6). Quartzite in contrast rose two units by ADD200, then dropped to one unit more acidic than the control at ADD 500, before returning to the level of the control at ADD600 but slowly sinking by one unit from ADD1000 to ADD1200. No correlation was noted with the soil water content, aside from a higher water content apparently enabling the body fluids to permeate the soil. It is currently unclear whether the pH of the soil affected the rate of decomposition.

## Discussion

### Temperature

While the meteorological temperatures differed significantly from buried temperatures in all samples, the ambient temperatures recorded on site showed no significant differences from the buried temperatures. This suggests that recordings of the ambient temperatures can be used for ADD calculations even for buried remains. Due to logistical limitations, the piglets were only buried at an approximate depth of 10–15 cm, which has been shown by Prangnell and McGowan [1] to be insufficient to noticeably alter temperatures from the surrounding environment. Furthermore, the frequent rainfall leading to a high soil water content would also ensure that the soil temperatures would match that of the environment [5]. Burial at greater depths (and in the ground, as opposed to in containers) would provide more insulation, which may in turn lead to results that differ dramatically from the present observations made regarding shallow burials.

### TBS progression patterns

The piglets interred in dolomite had the greatest variation in their TBS progression, with significant differences between almost all specimens interred in that soil type (Table 1A). The exact reasons for these differences are not readily apparent. It is possible that the properties of dolomite may expose interred remains to greater environmental variations than the other soil types. Forensic scientists should be cognisant of the variations of decomposing rates and patterns of remains buried in dolomite, as this will result in more variable estimated PMIs or necessitate estimating PMIs with larger ranges. In contrast, the quartzite specimens progressed relatively uniformly and displayed no significant differences in TBS progression (Table 1B). This would result in PMI estimations for remains buried in quartzite to be more reliable and narrower.

A general trend was observed in the TBS vs. ADD graphs, which all exhibited a sigmoid curve similar to previous research [22, 23]. Each curve indicated that the rate of decomposition over time exhibited three phases: an initial lagging phase, an intermediate phase of rapid decomposition, and a final plateauing phase (Figs. 2 and 3). However, the sigmoid pattern observed by Myburgh et al. [22] exhibited a rapid rate of decomposition, followed by a plateau and then a slowed rate of decomposition until skeletonization was reached. Subjectively grouping these phases of decomposition rates may be useful for obtaining a degree of error in PMI estimates, as each phase has progressively more minute changes in TBS (the observable variable). Further testing with a larger sample size is necessary to determine if these phases are repeatable and statistically relevant for use in forensic investigations. A logarithmic trend in TBS progression is widely noted in both buried [23, 26, 29] and surface remains [6, 22, 30] so there is merit in exploring this phenomenon further. The use of phases has not been explored as an option in previous literature, which provides a novel hypothesis for future studies.

Since dolomite and mine tailing specimens were the only comparison to exhibit significantly different TBS progressions from each other, this indicates that in all other soil comparisons, each soil’s unique combination of properties is not dissimilar enough to alter the rate of burial decomposition rates. Future burial studies do not need to be overly concerned with the type of soil being used in the study and can focus on variations in other burial-related factors. It is unknown what variances between dolomite and mine tailings resulted in the significant differences in TBS progression, which requires further investigation.

### Patterns of decomposition

Since dolomite has very low water retention (and consequently low water content) (Fig. 4), the environment in which the piglets were decomposing was neither so damp and anaerobic as to promote adipocere formation, nor so dry as to cause mummification [10]. Because of this, the piglets were rapidly and continuously colonised by maggots, leading to extensive soft tissue loss. Due to the effects of gravity, all moisture was drawn towards the inferior side of the piglet, leading to much more prolific maggot activity on the underside of the piglets. This led to the superior side appearing to be largely in an articulated state with some soft tissue present, while the underside was entirely defleshed and disarticulated due to minor disturbances. It is possible that the maggot activity (as well as the lower soil water content) may have led to very little tissue left to potentially form adipocere compared to the other buried remains.

The high water retention of quartzite led to adipocere formation on both sides of the piglets buried in this soil. While the maggots present had consumed a fair amount of soft tissue, leading to bony exposure on the limbs and head, the upper side of the piglets seemed to be largely intact. However, flipping the piglets revealed extensive decomposition on their undersides, leading to the torso breaking apart. It is suspected that maggot colonisation occurred early enough to allow for the destruction of a great deal of soft tissue before the onset of saponification and the resulting preservation.

The secondary research site where the mine tailing piglets were buried was frequented by visibly different Dipteran species, whose larvae were much larger and darker than those observed at the primary research site. Perhaps partly due to this, the rate of colonisation was exceptionally high. The rate of decomposition was similarly high, likely aided by the increased temperatures of the samples caused by the large numbers of metabolically active maggots [4]. A more detailed exploration of the impact of insect activity in buried remains buried in different soil types is outside the scope of the preset study. However, this is an important variable that requires further investigation in future studies.

TBS progressions were very similar for the dolomite and quartzite piglets, resulting in no significant differences between the two plots. However, it is important to note the frequency of adipocere formation in quartzite, which may lead to significant differences in a larger sample size. Further investigation into the use of the body’s upwards facing side compared to its downward facing side may be necessary to ascertain which of them is the more accurate in determining TBS and thereby ADD. The research carried out on decomposition within mine tailings is novel and will require further research to validate and broaden.

In general, the present study shows that the relatively high temperatures of the Gauteng province of South Africa, accompanied by the prevailing storms, lead to a subterranean environment that reduces small remains buried in shallow graves to skeletons with some residual saponified tissue within 1200 ADD (approximately two months). This indicates that estimating a post-mortem interval for remains that have been buried for more than a couple of months will be challenging.

### Soil pH

The soil pH fluctuations recorded by Haslam and Tibbett [11] are very similar to what was noted in the present study. Acidic soils (quartzite sourced from Gauteng, South Africa, and podsol sourced from Dorset county, England) both show a sharp upward pH shift soon after interment of decaying matter, followed by a prolonged decline (Figs. 6 ). The difference in pH trends between the two soils are that the total fluctuation of the quartzite pH was less than that of the podsol.

Alkaline soils (dolomite sourced from Gauteng, South Africa, and rendzina sourced from Dorset county, England) are less similar in their specific trends, but both fluctuate very little throughout the course of the experiment, barely deviating from their original pH by more than one unit. The limited pH fluctuation of dolomite may be explained by its high buffer capacity, allowing it to resist changes in pH from pollution or (in this case) decompositional fluids [17].

The more significant fluctuations of both the quartzite and the mine tailing pH indicate a conversely lower level of micronutrients, leading to a lower buffer capacity from free metal cations such as iron and magnesium [17]. This suggests that for investigations of burials in dolomite or similar soils, examining soil pH would not provide any useful information, while investigations of burials in quartzite or mine tailing might gain some information from testing soil pH. The pH trend line of the mine tailing soil does not have an analogue in the Haslam and Tibbett [11] study, as expected due to its chemically altered origin [18].

A more detailed understanding of the correlation between soil pH and the state of decomposition of a buried body will provide another data point in the estimation of PMI, which will be of great benefit to forensic investigators.

Since this was a proof-of-concept study, juvenile pigs were used for the sample according to the recommendations of Matuszewski et al. [7]. The novel results of this study prove that the soil properties do have a significant impact on the decomposition rate and patterns, and they provide a baseline for future studies to compare their results against. Future investigations require animal samples of larger size and weight and eventually validation of the results using human remains for forensic realism and application in official forensic investigations.

## Conclusion

This study featured a multifaceted approach at investigating the effects of different soil types on the decomposition of buried remains. The study produced TBS-ADD equations to estimate the PMI of remains buried in quartzite, dolomite, and gold mine tailing soil from TBS and ADD data with very high correlation coefficients. This suggests that the equations hereby generated are potentially reliable for preliminary and supplementary use to estimate the PMI of buried remains, but validation and refinement are necessary to yield the most accurate results and to generate equations that may be used for human remains. It was shown that while dolomite pH fluctuated only very slightly, quartzite and mine tailing pH values changed notably through the course of the experiment.

The validity of this data is impacted by the use of containers filled with soil, as opposed to “natural” interment in the earth, and further research with complete interment should be conducted. Furthermore, the small sample size required the use of non-parametric statistical analysis; future studies with greater manpower should try to use at least 30 specimens, preferably adults, to more closely simulate the decomposition of an adult human.

## Data Availability

Data sharing is not applicable to this article as no datasets were generated or analyzed during the current study.

## Declarations

### Disclaimers

The authors conceived this study, and performed the data collection, analysis and interpretation, and the writing of this article. The manuscript, data, figures, and tables have not been previously published and are not under consideration elsewhere.
